# MicroRNA Let-7f Inhibits Tumor Invasion and Metastasis by Targeting MYH9 in Human Gastric Cancer

**DOI:** 10.1371/journal.pone.0018409

**Published:** 2011-04-18

**Authors:** Shuli Liang, Lijie He, Xiaodi Zhao, Yu Miao, Yong Gu, Changcun Guo, Zengfu Xue, Weijia Dou, Fengrong Hu, Kaichun Wu, Yongzhan Nie, Daiming Fan

**Affiliations:** 1 State Key Laboratory of Cancer Biology and Institute of Digestive Diseases, Xijing Hospital of Digestive Diseases, Xi'an, China; 2 Department of Nephrology, Xijing Hospital, The Fourth Military Medical University, Xi'an, China; 3 Department of Gastroenterology, The Affiliated Hospital of Ningxia Medical University, Yinchuan, China; University of Bergen, Norway

## Abstract

**Background:**

MicroRNAs (miRNAs) are important regulators that play key roles in tumorigenesis and tumor progression. A previous report has shown that let-7 family members can act as tumor suppressors in many cancers. Through miRNA array, we found that let-7f was downregulated in the highly metastatic potential gastric cancer cell lines GC9811-P and SGC7901-M, when compared with their parental cell lines, GC9811 and SGC7901-NM; however, the mechanism was not clear. In this study, we investigate whether let-7f acts as a tumor suppressor to inhibit invasion and metastasis in gastric cancers.

**Methodology/Principal:**

Real-time PCR showed decreased levels of let-7f expression in metastatic gastric cancer tissues and cell lines that are potentially highly metastatic. Cell invasion and migration were significantly impaired in GC9811-P and SGC7901-M cell lines after transfection with let-7f-mimics. Nude mice with xenograft models of gastric cancer confirmed that let-7f could inhibit gastric cancer metastasis in vivo after transfection by the lentivirus pGCsil-GFP- let-7f. Luciferase reporter assays demonstrated that let-7f directly binds to the 3′UTR of MYH9, which codes for myosin IIA, and real-time PCR and Western blotting further indicated that let-7f downregulated the expression of myosin IIA at the mRNA and protein levels.

**Conclusions/Significance:**

Our study demonstrated that overexpression of let-7f in gastric cancer could inhibit invasion and migration of gastric cancer cells through directly targeting the tumor metastasis-associated gene MYH9. These data suggest that let-7f may be a novel therapeutic candidate for gastric cancer, given its ability to reduce cell invasion and metastasis.

## Introduction

Gastric cancer (GC) is the most common gastrointestinal malignancy in East Asia, Eastern Europe, and parts of Central and South America, and is the second leading cause of cancer-related deaths [1]. Widespread metastasis has been a major reason for the dismal outcome of GC patients. Metastasis is a complex, multi-step process whereby cancer cells migrate from the primary neoplasm to a distant location [2]. The process starts when primary tumor cells invade adjacent tissue, followed by cells entering into the blood stream (intravasation), translocating through the vasculature, exiting from blood vessels (extravasation) into the surrounding tissue parenchyma, initiating micrometastases and finally proliferating to form macroscopic secondary tumors [3]. One of the critical regulators that involved in this process is a microRNA (miRNA) [4].

MicroRNAs (miRNAs) are a class of endogenous and small non-coding regulatory RNAs, which regulate genes at the post-transcriptional level [5]. Mature miRNAs can be transcribed by RNA polymerase II and are generated from the sequential processing of primary miRNA transcripts by Drosha and Dicer; they then serve as posttranscriptional regulators of gene expression through complementary base pairing to messenger RNAs [6]. Many reports show that miRNAs play key roles in various biological processes, including cell differentiation, proliferation, apoptosis, stress resistance, fat metabolism, tumorigenesis, and tumor metastasis [7], [8], [9].

The let-7 family is a conserved family of miRNAs. Let-7 was originally observed in the nematode Caenorhabditis elegans [10], and fourteen members have been found to date [11]. Recently, the expression levels of many let-7-family members were found to be reduced in a variety of cancers. For example, let-7 is downregulated in lung cancer, melanoma, and head and neck squamous carcinoma, while overexpression of let-7 can inhibit cancer cell growth [12], [13], [14], [15], [16], [17], [18]. Several oncogenes, such as RAS, MYC, and HMGA2, are direct targets of let-7 [19], [20], [21]. Let-7f is one member of the let-7 family. Previous studies have shown that let-7f is up-regulated in primary breast cancer and promotes angiogenesis [22]; downregulation in PAH [23] was caused by chronic hypoxia or monocrotaline in rats, and the level was decreased in plasma vesicles of NSCLC patients [24]. In addition, let-7f can affect cell proliferation by targeting Kallikrein-related peptidases (KLKs), a family of serine proteases that have been shown to be dysregulated in several malignancies, including ovarian cancer [25].

Our lab have previously identified a group of differentially expressed miRNAs between GC9811 and GC9811-P cells through high-profile microRNA chip analyses that contain let-7f [26]. During the further characterization of let-7f in various cancer cell lines, we found that let-7f functions as a metastasis suppressor. The enhanced expression of let-7f can suppress GC cell invasion and migration in vitro and in vivo. By bioinformatics analysis, we identified that MYH9, which encodes a myosin IIA heavy chain involved in the promotion of cancer cell migration or invasion, as a putative let-7f target. Subsequent experiments confirmed that let-7f can downregulate myosin IIA expression by targeting the 3′UTR of MYH9, which provides a possible target for GC treatment.

## Materials and Methods

### Tissue collection

Primary gastric tumor tissues, adjacent non-tumor gastric tissues and distant metastatic gastric tissues were obtained from patients who underwent surgery at the Xijing Hospital of Digestive Diseases in Xi'an, China. All samples were clinically and pathologically shown to be correctly labeled. Patients offering samples for the study signed informed consent forms.

### Cell culture

The human gastric cancer cell lines GC9811, GC9811-P, SGC7901-NM, SGC7901-M were conserved in our own laboratory and were cultured in RPMI1640 (HyClone), supplemented with 10% fetal bovine serum (FBS; GIBCO ), 100 units/ml penicillin, and 0.1 mg/ml streptomycin at 37°C in a humidified 5% carbon dioxide incubator.

### RNA extraction and real-time PCR

qRT-PCR was performed to determine the expression levels of potential let-7f target genes. Total RNA was extracted from tissues or cultured cells using TRIZOL reagent (Invitrogen Life Technologies). Complementary DNA (cDNA) was generated by using a TaqMan Reverse Transcription Kit (Applied Biosystems). Real-time PCR analyses were performed with a TaqMan Micro-RNA Assay kit (Applied Biosystems). Primer of let-7f was purchased from Ambion(MI0000437). Primer of MYH9 sequence was designed using Primer Express Software (Version 1.5). The primer-MYH9 sequence :(Forward) 5′AGAGCTCACGTGCCTCAACG3′ (Reverse) 5′TGACCACACAGAACAGGCCTG3′.All protocols were carried out according to the manufacturer's instructions. The expression level of let-7f was normalized to 5S(Forward:5′GATTGAATCGAGCACCAGTTAC3′;

Reverse: 5′GTCTACGGCCATACCACCCTGAAC3′). The expression level of MYH9 was normalized to18S(Forward:5′CGGCTACCACATCCAAGGAA3′;

Reverse: 5′GCTGGAATATCCGCGGCT3′).PCR and data collection were performed on an iCycler (Bio-Rad). Each sample was run in triplicate.

### Vector constructs and Lentivirus Production

The pri-let-7f sequence was constructed as follows: (Forward) hsa-let-7f-1-Xho I-F GGGCCCGCTCTAGACTCGAGATATTTGCATGTCGCTATGTG, (Reverse) hsa-let-7f-1- BamH I-R CGCGGCCGCCTAATGGATCCAAAAAAGGCACAGTCGAGGCTGATC. The sequence was amplified and cloned into the pGCsil-GFP Vector (GENECHEM) to generate pGCsil-GFP- let-7f. Negative control was pGC FU-RNAi-NC-LV. Virus packaging was performed in HEK 293T cells after the cotransfection of 20 µg pGCsil-GFP-let-7f vector with 15 µg of the packaging plasmid pHelper 1.0 Vector and 10 µg of the envelope plasmid pHelper 2.0 Vector using Lipofectamine 2000 (Invitrogen). Viruses were harvested 48 h after transfection, and viral titers were determined.

### Oligonucleotide construction

let-7f mimics, let-7f inhibitor and negative control siRNA oligonucleotides were chemosynthesized (Shanghai GenePhama Co., Ltd). The oligonucleotides used in these studies were has-let-7f mimics: 5′UGAGGUAGUAGAUUGUAUAGUU3′and 5′CUAUACAAUCUACCUCAUU3′;

Mimics negative control: 5′UUCUCCGAACGUGUCACGUT3′and5′ACGUGACACGUUCGGAGAATT3′, has-let-7f inhibitor: 5′AACUAUACAAUCUACUACCUCA3′. MircoRNA inhibitor negative control:5′CAGUACUUUUGUGUAGUACAA3′.

### Cell transfection

Cells were cultured to 80% to 90% confluence after being seeded into 6-well plates and were transfected with Lipofectamine 2000 (Invitrogen, Carlsbad, CA) according to the manufacturer's instructions. For transient transfection, cells in each well of a 6-well plate were transfected with 12.5 µl miRNA inhibitor or 7.5 µl miRNA mimic oligonucleotides. After 48 h of transfection, cells were harvested for further experimentation. For stably transfected cells, cells were transfected with lentivirus at 30%–50% confluency. Target cells (1×10^4^), including GC 9811-P and SGC7901-M cells, were infected with 1×10^6^ recombinant lentivirus-transducing units in the presence of 6 µg/mL polybrene (GENECHEM).

### Invasion assay

The invasive ability of the cells was assayed using Transwells (8-µm pore size, Corning Costar Corp). The Transwells were put into the 24-well plates. First, 0.1 ml Matrigel(50 µg/ ml, BD Biosciences) was added onto the plate surface and incubated for 2 h, and then the supernatant was removed. Freshly trypsinized and washed cells (GC9811, GC9811-p, SGC7901-NM, SGC7901-M) were suspended in RPMI1640 containing 1% fetal bovine serum. Then 100 µl of the cell suspension (1×10^5^ cells) was added to the upper chamber of each insert that was coated with Matrigel. Next, 450 µl of RPMI1640 containing 10% fetal bovine serum was added into the lower compartment, and the cells were allowed to invade for 24 h–48 h at 37°C in a 5% CO_2_ humidified incubator. After incubation, the cells were fixed with 95% absolute alcohol and stained with crystal violet. Cells on the upper surface of the filter were removed with the cotton swab and the cells that had invaded into the bottom surface of the filter were counted and imaged under an inverted microscope (Olympus Corp. Tokyo, Japan) at ×200 magnification over ten random fields in each well. Each experiment was performed in triplicate.

### Cell migration

The ability of GC9811, GC9811-P, SGC7901-NM, and SGC7901-M cells to migrate was detected using Transwells [8-µm pore size, Corning Costar Corp]. The Transwells were put into the 24-well plates. Freshly trypsinized and washed cells were suspended in RPMI1640 containing 1% fetal bovine serum. 5×10^4^ cells /well were placed in the top chamber of each insert (BD Biosciences, NJ), with the non-coated membrane. 450 µl of RPMI1640 containing 10% fetal bovine serum was added into the lower chambers. After incubating for 24 h–48 h at 37°C in a 5% CO_2_ humidified incubator, cells were fixed with 95% absolute alcohol and stained with crystal violet. The cells in the inner chamber were removed with a cotton swab and the cells attached to the bottom side of the membrane were counted and imaged under an inverted microscope (Olympus Corp. Tokyo, Japan) at ×200 magnification over ten random fields in each well. Each experiment was performed in triplicate.

### In Vivo metastasis assay

For the in vivo metastasis assays, the stable cell lines GC9811-P and SGC7901-M were harvested from tissue culture flasks after transfection with the lentivirus pGCsil-GFP- let-7f and control pGCsil-GFP using trypsin and washed three times with PBS. Then 2×10^6^ cells were suspended in 0.2 ml serum-free RPMI1640 for each mouse (six in each group, Fale BALB/c-nu/nu, 6–8 weeks age), and the cells were injected into the tail vein. After four weeks of injection, the mice were sacrificed. Liver tissue was observed with the naked eye, and the number of visible tumors in the liver surface was counted. The liver tissues were divided into serial sections, fixed with phosphate-buffered neutral formalin, stained with hematoxylin and eosin and examined histologically. Nude mice were manipulated and cared for according to NIH Animal Care and Use Committee guidelines in the Experiment Animal Center of the Fourth Military Medical University (Xi'an, Shanxi Province, P.R. China).

### Luciferase Reporter Assay

To investigate whether MYH9 expression was regulated by let-7f, a dual-luciferase reporter assay was performed. The 3′ UTR of MYH9 containing let-7f binding site was amplified by PCR. Amplification of MYH9 used the following primers: (Forward) XhoIF: 5′CCGctcgagGCCTCTTCTCCTGCAGCCTG 3′ (Reverse) NotIR: 5′ATAAGAATgcggccgcTCGTAGCACATGGTTCTCTTTATTG 3′

As a negative control, the mutated binding site of the 3′-UTR sequence (using the reverse complement of the binding site) was amplified using the primers: (Forward) mutlet7MYH9F: 5′TTGCAATCACACGTGGTGTGGAGTCACACCTCTGCCCCTTGG3′ (Reverse) mutlet7MYH9R: 5′CCAAGGGGCAGAGGTGTGACTCCACACCACGTGTGATTGCAA 3′. Products were reclaimed via agarose gel electrophoresis, and cloned into the luciferase reporter PsiCHECK vector (Promega, Madison, WI). All constructs were verified by sequencing. Log phase GC9811-P cells were seeded into 24-well plates and cotransfected with Let-7f inhibitors or control, and luciferase reporters using Lipofectamine™ 2000 (Invitrogen), following the instructions. After 48 h of incubation, firefly and Renilla luciferase activity was measured with the dual luciferase reporter assay system (Promega).

### Western Blot

Cells were washed twice with ice-cold PBS and scraped into RIPA buffer (50 mM Tris–HCl pH 7.4, 1% (v/v) Triton X-100, 1 mM EDTA, 1 mM leupeptin, 1 mM phenylmethylsulfonyl fluoride, 10 mM NaF, 1 mM Na3VO4) with freshly added protease inhibitor cocktail (Roche) for 15 min on ice, then centrifuged at 13,000 rpm for 10 min and the supernatants were collected. Ten micrograms of each sample was resolved using 6% SDS-PAGE and transferred onto a nitrocellulose membrane (Bio-Rad, Hercules, CA). The bands were incubated with 10% nonfat dry milk in Tris-buffered saline–0.1% Tween-20 to block the nonspecific binding sites and incubated with a primary antibody: rabbit polyclonal anti-human myosin IIA (diluted 1∶500; Cell Signaling Technology) overnight at 4°C. After rewarming and repeated washing three times,15 min for each one, the membranes were incubated with horseradish-peroxidase-conjugated anti-rabbit secondary antibody (Santa Cruz Biotechnology), diluted 1∶2000 for two hours at room temperature. The bands were detected using an enhanced chemiluminescence system (Amersham Biosciences).

### Immunohistochemistry

Two tissue arrays were purchased from SHANXI CHAOYING BIOTECHNOLOGY CO.,LTD. One was gastric cancer tissue and matched adjacent normal stomach tissue, the other was gastric cancer tissue and matched lymph node metastasis .Tissue arrays were dewaxed in 60°C for 2 h, high temperature antigen recovery for 10 mins, rehydrated, incubated in 10% normal goat serum for 1 h, then incubated with rabbit polyclonal anti-human myosin II A(diluted 1∶100; Cell Signaling Technology) overnight at 4°C. The slides were washed in PBS three times for 5 min each. The tissues were incubated in goat anti-rabbit serum (DAKO) for 1 h, rinsed with PBS. Sections were detected using DAB and counterstained with hematoxylin. The staining intensity was scored using a four step scale (0, 1+, 2+, or 3+) and the percentage of positive cells was estimated by three different pathologists (0 = none, 1 = <1%, 2 = 1–10%, 3 = 10–30%, 4 = 30–70%, 5 = >70% of tumor cells). Total average scores were then grouped into four categories: negative (no detectable staining when evaluated compared to nonspecific background staining, weak (+), moderate(++)or strong (+++) and compared using Mann-Whitney test.

### Statistical Analysis

Student's t-test (two-tailed), One-way ANOVA and Mann-Whitney test were employed to analyze the in vitro and in vivo data using SPSS 12.0 software (Chicago, IL, USA). P value<0.05 was defined as statistically significant. * *P*<0.05; **P<0.01.

## Results

### The level of let-7f expression was frequently decreased in human GC metastatic cell lines and tissues

**Figure 1:**
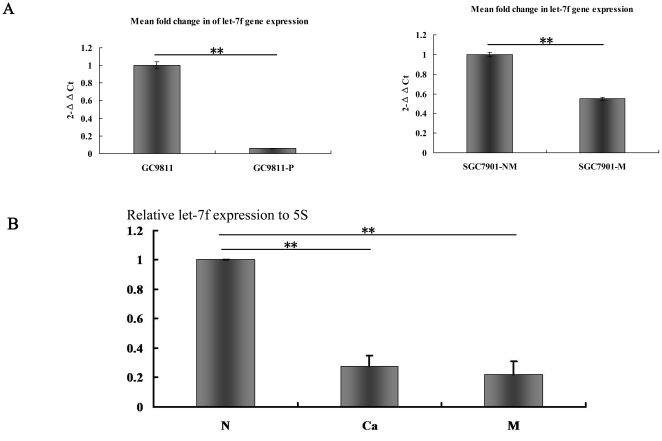
Let-7f expression in gastric cancer tissues and cells measured by real-time PCR. (A) The mean fold-changes of let-7f gene expression in GC9811 and GC9811-P(right). The mean fold-changes of let-7f gene expression in SGC7901-NM and SGC7901-M(left). Student's t-test, n = 3. (B) The relative expression of let-7f to 5S. * *P*<0.05. ** *P*<0.01. one-way ANOVA, n = 8. Real-time PCR show that the relative expression of let-7f in eight adjacent non-cancerous gastric tissue samples, and in gastric cancer cell lines GC9811, SGC7901-NM is higher than that in their primary gastric cancer and distant gastric metastasis specimens and the high potential metastasis gastric cancer cell GC9811-P and SGC7901-M.Each sample was analyzed in triplicate and normalized to 5S. Fold change was calculated by 2-*ΔΔ*Ct.

We have examined the mRNA expression levels of let-7f in two pairs of human gastric cancer cell lines, GC9811, GC9811-P and SGC7901-NM, SGC7901-M. As shown in **Figure 1A**, expression of let-7f was lower in the GC cells, GC9811-P and SGC7901-M, with high metastatic potential, while let-7f was more highly expressed in the GC cells, GC9811 and SGC7901-NM, with low metastatic potential. To further validate the role of let-7f in gastric cancer cells metastasis, we compared the expression levels of let-7f in fresh human gastric cancer tissue specimens with metastases from eight individuals (Table S1), to normal gastric tissues (NG), primary gastric cancer tissues (GC) and distant metastatic gastric cancer tissues (MC), respectively, by real-time PCR. Intriguingly, there was remarkable downregulation of let-7f in MC and GC, compared to let-7f expression in NG (**Figure 1B**). We observed that either the loss of or decreased expression of let-7f in tumors and their metastatic tissues resulted in enhanced metastasis in gastric cancer tissues. The results all suggested that the expression of let-7f was negatively correlated very closely with decreased GC metastases and might play a vital role in pathological processes. We have thus provided clinical and cellular conceptual evidence for a metastatic switch in cancer development that suggests a key role for the expression of let-7f in cancer development.

### Let-7f inhibited the invasive and metastatic abilities of GC cells in vitro

**Figure 2:**
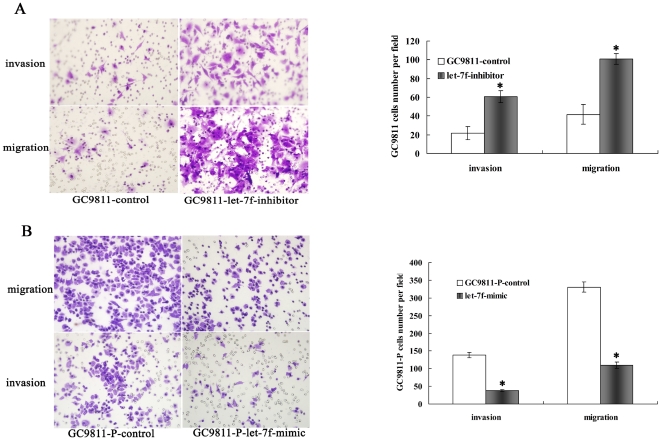
Effect of let-7f on tumor cell invasion and metastasis of GC9811and GC9811-P Cells. (A) Invasion and migration assay. Representative fields of invasive (up) or migration (down) cells on the membrane(left). (magnification of ×200). Average invasive or migration cell number per field (right). The invasive or migration cell number of GC9811 cells transfected with let-7f-inhibitors is drastically increased than that transfected with negative control. *P<0.05, Student's t-test, n = 10. (B). The invasive or migration cell number of GC9811-P Cells transfected with let-7f-mimics is drastically decreased than that transfected with negative control. *P<0.05, Student's t-test, n = 10.

**Figure 3:**
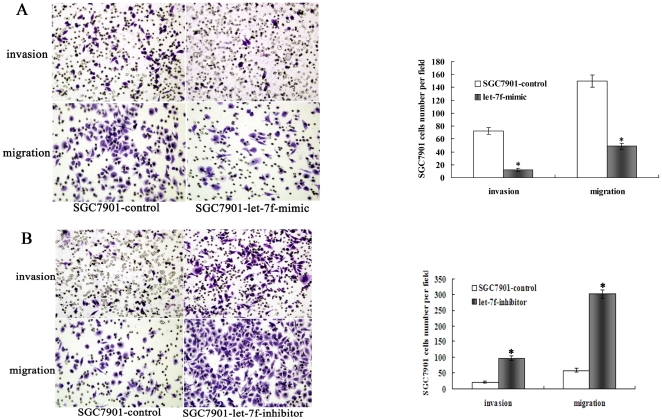
Effect of let-7f on tumor cell invasion, metastasis of SGC7901-NM and SGC7901-M cells. (A) Invasion and migration assay. Representative fields of invasive (up) or migration (down) cells on the membrane(left). (magnification of ×200). Average invasive or migration cell number per field (right). The invasive or migration cell number of SGC7901-NM transfected with let-7f-inhibitors is drastically increased than that transfected with negative control. *P<0.05,Student's t-test, n = 10. (B) The invasive or migration cell number of SGC7901-M Cells transfected with let-7f-mimics is dramatically decreased than that transfected with negative control. *P<0.05, Student's t-test, n = 10.

Because let-7f acts as an agitator in invasion and metastatic processes, we investigated the effects of decreased let-7f on less-metastatic cell lines and increased let-7f on more-metastatic cell lines. To accomplish this, short interfering RNA (siRNA) oligonucleotides of let-7f were constructed and introduced into GC9811 cells and SGC7901 -NM cells for metastasis assays in vitro, as GC9811-let-7f-siRNA cells, SGC7901-NM-let-7f-siRNA cells and control cells. Concurrently, mimic-let-7f and negative control oligonucleotides were also constructed and introduced into GC9811-P cells and SGC7901-M cells for metastasis assays in vitro, as GC9811-P-let-7f-mimic cells, SGC7901-N-has let-7f-mimic cells and control cells. Depletion of let-7f significantly impaired the ability of GC9811 cells to migrate and invade through the matrigel-coated membranes or the non-matrigel-coated membranes towards serum-containing medium in a modified Boyden chamber assay (**Figure 2A**). Increased expression of let-7f significantly suppressed the ability of GC9811-P cells to migrate and invade through matrigel-coated membranes or non-matrigel-coated membranes towards serum-containing medium in a modified Boyden chamber assay (**Figure 2B**), when compared with the control cells. Similar results were found in SGC7901-NM cells (**Figure 3A**) and SGC7901-M cells (**Figure 3B**), while let-7f knockout in GC cell lines resulted in higher invasion and migration rates.

### Let-7f inhibits tumor invasion and metastasis in a nude mouse xenograft model

**Figure 4:**
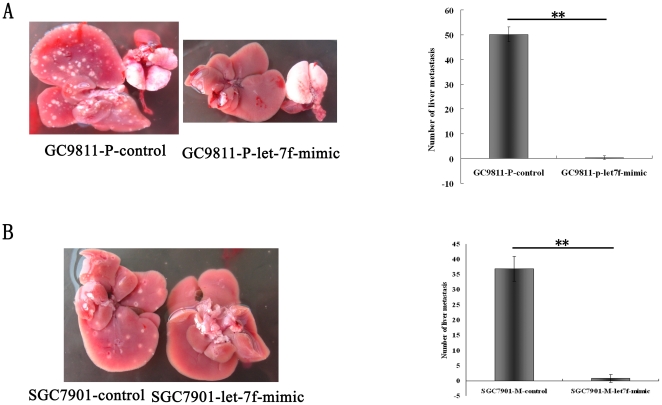
In vivo metastasis assay. GC9811-P and SGC7901-M cells were transfected with lentivirus pGCsil-GFP-let-7f or negative-control lentivirus pGCsil-GFP, and then injected into nude mice via the tail vein, as described in Materials and Methods. Animals were sacrificed 4 weeks after injection. (A) Representative anatomical photos of lives from mice injected with GC9811-P- pGCsil-GFP or GC9811-P- pGCsil-GFP- let-7f(left). The mean number of visible tumor nodules in liver (right). P<0.01. Student's t-test, n = 6. (B) Representative anatomical photos of lives from mice injected with SGC7901-M - pGCsil-GFP or SGC7901-M - pGCsil-GFP- let-7f(left). The mean number of visible tumor nodules in liver(right). P<0.01. Student's t-test, n = 6.

GC9811-P or SGC7901-M tumor cells transfected with the lentivirus pGCsil-GFP- let-7f or negative control pGCsil-GFP were injected into nude mice and the mice were sacrificed four weeks after the inoculations. The number of metastatic nodi was dramatically reduced in the nude mice injected with the pGCsil-GFP- let-7f transfected cells, when compared to the negative controls (**Figure 4A, 4B**). These data provide strong evidence that let-7f can inhibit tumor invasion and metastasis in vivo.

### Let-7f downregulates myosin IIA expression by directly targeting its 3′ UTR

**Figure 5:**
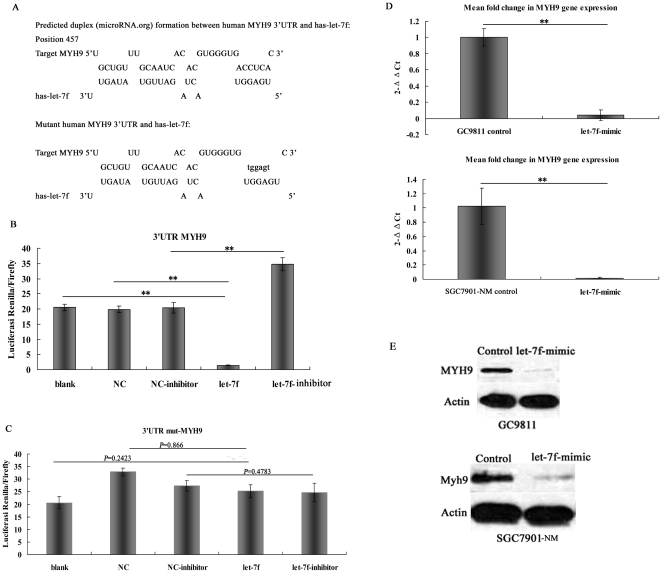
let-7f directly targets MYH9. (A) Predicted duplex formation between human MYH9 3′UTR (top) and has-let-7f (bottom) and the sequence of the let-7f-binding site within the MYH9 3′UTR and mutant (mut) MYH9 3′UTR. Luciferase activity of the MYH9 3′UTR (B) or mut MYH9 (C) 3′UTR reporter gene in GC9811 cells infected with the let-7f, let-7f-inhibitor, blank or empty mimic oligo. The assays showed that luciferase activities in the group were significantly decreased compared to those of the mutant and negative control groups. * P<0.05. (D) The expression of MYH9 was analyzed by qRT-PCR. MYH9 was decreased in GC9811 cells and SGC7901 cells transfected with let-7f-mimics compared to GC9811 cells and SGC7901 cells transfected with let-7f -negative control. (E) let-7f suppresses the endogenous protein levels of MYH9, as detected by Western blot. Stable transduced SGC7901-NM and GC9811 cells ectopically expressing let-7f were used for Western blot analysis. Effect of let-7f on MYH9 in GC9811 cells (top). Effect of let-7f on MYH9 in SGC7901-NM cells (bottom).

To further explore the mechanism by which let-7f suppresses GC invasion and metastasis, we analyzed potential downstream tumor metastasis-related target genes in silico. We focused on let-7f target genes, especially those genes that were migration and/or invasion-related, and found that myosin IIA, involved in the promotion of cancer cell migration or invasion, might be the target gene of let-7f. In an effort to determine whether myosin IIA is regulated by let-7f through direct binding to its 3′ UTR, we constructed full-length wild-type and mutant fragments of MYH9 mRNA 3′ UTR, and inserted them into the region immediately downstream of a luciferase reporter gene (**Figure 5A**). Subsequently, let-7f mimic oligos were co-transfected with different luciferase 3′ UTR constructs into GC9811-P cells. We found that let-7f decreased the relative luciferase activity in the wild-type 3′ UTR of MYH9 (**Figure 5B**). However, luciferase activity did not drop sharply in the UTRs with mutant binding sites, when compared to the mut-type counterparts (**Figure 5C**). These data support that MYH9 is direct target of let-7f.

**Figure 6:**
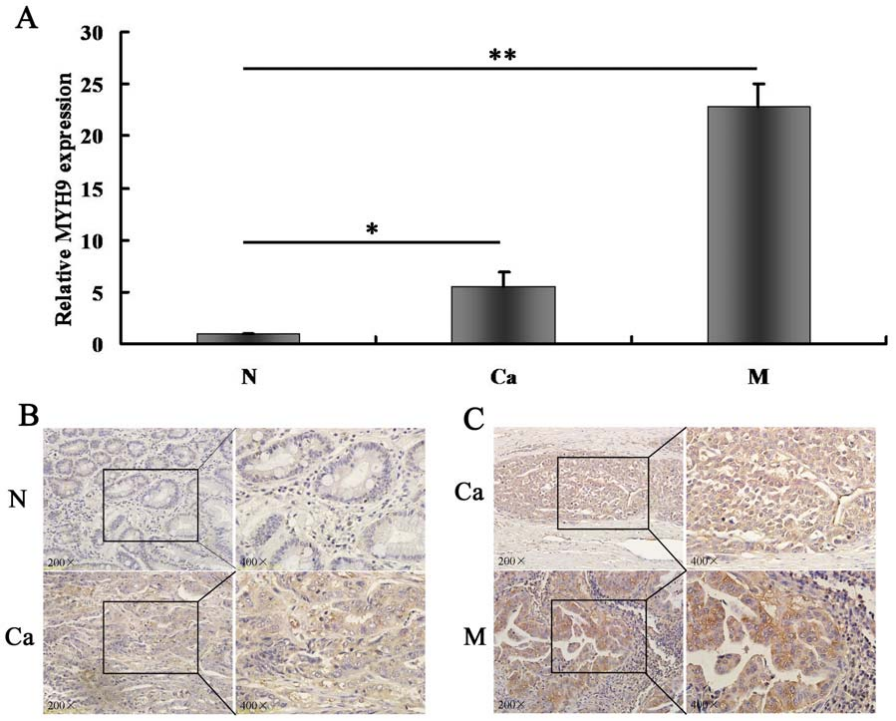
The expression of MYH9 in gastric tumor specimens. (A) Real-time PCR show that the relative expression of MYH9 in eight distant gastric metastasis specimens is higher than that in their primary gastric cancer and adjacent non-cancerous gastric tissue samples. Each sample was analyzed in triplicate and normalized to 18S. (B) Expression of MYH9 in primary gastric cancer (Ca) and its adjacent normal stomach tissue (N) by IHC. (C) Expression of MYH9 in primary gastric cancer (Ca) and its matched lymph node metastasis tissue (M) by IHC.

RT-PCR showed that the expression of MYH9 in GC9811 cells and SGC7901-NM cells transfected with let-7f-mimics is downregulated compared to those cells transfected with control constructs (**Figure 5D**). Western blotting results showed expression of myosin IIA in GC9811 and SGC7901-NM cells transfected with let-7f-mimics are down-regulated compared to those transfected with negative control (**Figure 5E**). Furthmore, Real-time PCR shows that mRNA relative expression of MYH9 in GC tissues and distant metastatic tissue are down-regulated compared with their normal adjacent non-cancerous specimens (**Figure 6A**). Immunohistochemistry showed that the expression of myosin IIA in GC lymph node metastasis is up-regulated compared with its primary GC tissue (**Table 2** **;** **Figure 6C** **; Figure S1B**), and it is up-regulated in GC tissue compared with its matched adjacent normal stomach tissue (**Table 1** **;** **Figure 6B** **; Figure S1A**).These data demonstrate that let-7f can down-regulate the mRNA expression of MYH9 and can repress protein translation of it.

**Table 1:** Myosin IIA expression in gastric cancer tissue and matched adjacent normal stomach tissue.

	Negative(−)	Weak(+)	Moderate(++)	Strong(+++)	P
N	9 of 40(22.5%)	22 of 40(55%)	9 of 40(22.5%)	0 of 40(0)	
Ca	3 of 40(7.5%)	20 of 40(50%)	15of 40(37.5%)	2 of 40(5%)	<0.05

Note: Ca-gastric cancer tissue; N-matched adjacent normal stomach tissue.

The expression of myosin IIA is higher in primary gastric cancer tissue compared with matched adjacent normal stomach tissue (P<0.05).

**Table 2:** Myosin IIA expression in gastric cancer tissue and matched lymph node metastasis.

	Negative(−)	Weak(+)	Moderate(++)	Strong(+++)	P
Ca	4 of 40(10%)	21 of 40(52.5%)	12 of 40(30%)	3 of 40(7.5%)	
M	6 of 40(15%)	9 of 40(22.5%)	7 of 40(17.5%)	18 of 40(45%)	<0.05

Note: Ca-gastric cancer tissue; M-matched lymph node metastasis.

The expression of myosin IIA is higher in lymph node metastasis compared with primary gastric cancer tissue (P<0.05).

## Discussion

In the present study, we demonstrated that let-7f expression in metastatic GC cell lines was downregulated when compared to let-7f expression in non-metastatic GC cells. Ectopic expression and siRNA knockdown of let-7f confirmed its invasion-suppressing activity in vitro and in vivo. Moreover, we show that MYH9 is a direct target for let-7f and this let-7f mediated suppression of MYH9 is dependent on its 3′-UTR. Therefore, these results highlight the significance of let-7f as a tumor suppressor in cell invasion and metastasis by targeting MYH9 in gastric cancer.

Recent studies show that miRNAs may act as activators or inhibitors of tumor metastasis [27]. For example, microRNA-10b [28], miR-373 and miR-520c [29], [30] stimulated cancer cell migration and invasion in breast cancer. Additionally, miR-155 can promote tumor invasion and metastasis in breast cancer by downregulating its target, RhoA [31] and promote tumor invasion and metastasis in pancreas duct carcinoma by repressing the expression of TP53INP1 [32]. The miRNA-200 family (miRNA-200a, miRNA-200b, miRNA-200c, miRNA-141 and miRNA-429) can inhibit tumor invasion and metastasis by regulating the EMT [33]. MiR-126 was found to inhibit cell adhesion, migration and invasion partially through the suppression of CRK in an in vitro model of non-small-cell lung carcinoma [34]. It has been shown that miR-29c is significantly reduced in highly invasive and metastatic nasopharyngeal carcinoma [35]. MiR-218 inhibits the invasion and metastasis of gastric cancer by targeting the Robo1 receptor [26]. Although many studies have been done to investigate the mechanism of miRNA and tumor metastasis, the mechanism was not clear. Most importantly, few studies have been done on the mechanisms of GC metastasis regulation by microRNA.

The let-7f gene is located at 9q22.3, and is involved in a variety of physiological and pathological processes, including angiogenesis [36], immunocyte differentiation [37], replicative senescence [38], growth arrest [39], pulmonary arterial hypertension and carcinogenesis [23]. Let-7f is downregulated in several malignancies. A highly characterized example is renal cell carcinoma, in which let-7f downregulation led to a significant decrease in kallikrein (KLK) expression [40]. KLKs can also be targeted by let-7f in ovarian cancer [25]. In papillary thyroid cancer, reduced expression of let-7f might be an essential molecular event in RET/PTC malignant transformation [41]. In the case of primary breast cancer, however, let-7f was upregulated when compared to normal adjacent tumor tissues [22]. In our study, we demonstrated that let-7f is also downregulated in MC tissues and GC cell lines with high metastatic potential, and we further explored the mechanism by which let-7f reduced tumor invasion and metastasis.

MYH9 is the gene for non-muscle myosin heavy chain IIA (NMHCIIA), whose mutations are responsible for a complex disorder named MYH9-related disease, characterized by a combination of different phenotypic features [42]. NMMHC-IIA, a 1960 amino acid polypeptide, with a translated molecular weight of 220 kDa, is a conventional, non-sarcomeric myosin expressed in most cells and tissues. Its functions include roles in cytokinesis, cell motility, and maintenance of cell shape [43]. Many studies suggest that MYH9/NMHC-IIA has a key role in tumor cell invasive behavior.For example, the EGF-dependent phosphorylation of the myosin-IIA heavy chain has a direct role in mediating motility and chemotaxis in MDA-MB-231 human breast cancer cells [44]. Myosin IIA appears to be a major cellular target of mts1 [45], the small calcium-binding protein metastasin-1, and co-localizes with mts1 to the leading edge of migrating cancer cells [46]; mts1 influences both the assembly behavior of myosin IIA and its phosphorylation [47], [48]. A recent report implicates MYH9 (NMHC-IIA) as a target of SRF, which contributes to invasion and metastasis in breast cancer [49]. Our data show that MYH9 is the direct target of let-7f, which inhibited invasion and metastasis by binding the 3′UTR of MYH9, which resulted in the downregulation of myosin IIA expression.

In conclusion, our study demonstrates that let-7f can suppress the invasion and metastasis of gastric cancer by directly binding the 3′UTR of MYH9, its target. Though there is still much to learn about the role of let-7f in gastric cancer tumorigenesis, let-7f provides us with a new potential target for gastric cancer treatment.

## Supporting Information

Figure S1 **The expression of MYH9 in gastric tumor specimens.** (A) Expression of MYH9 in primary gastric cancer (Ca) and its adjacent normal stomach tissue (N) by IHC. (B) Expression of MYH9 in primary gastric cancer (Ca) and its matched lymph node metastasis tissue (M) by IHC.
(TIF)

Table S1 **Clinicopathologic features in 8 fresh gastric tumor samples.**
(DOC)
