# Supplementary figures and images for: MicroRNA Let-7f Inhibits Tumor Invasion and Metastasis by Targeting MYH9 in Human Gastric Cancer
Source: PLoS One. 2011 Apr 18;6(4):e18409. doi: 10.1371/journal.pone.0018409 (PMC3078939; doi:10.1371/journal.pone.0018409)

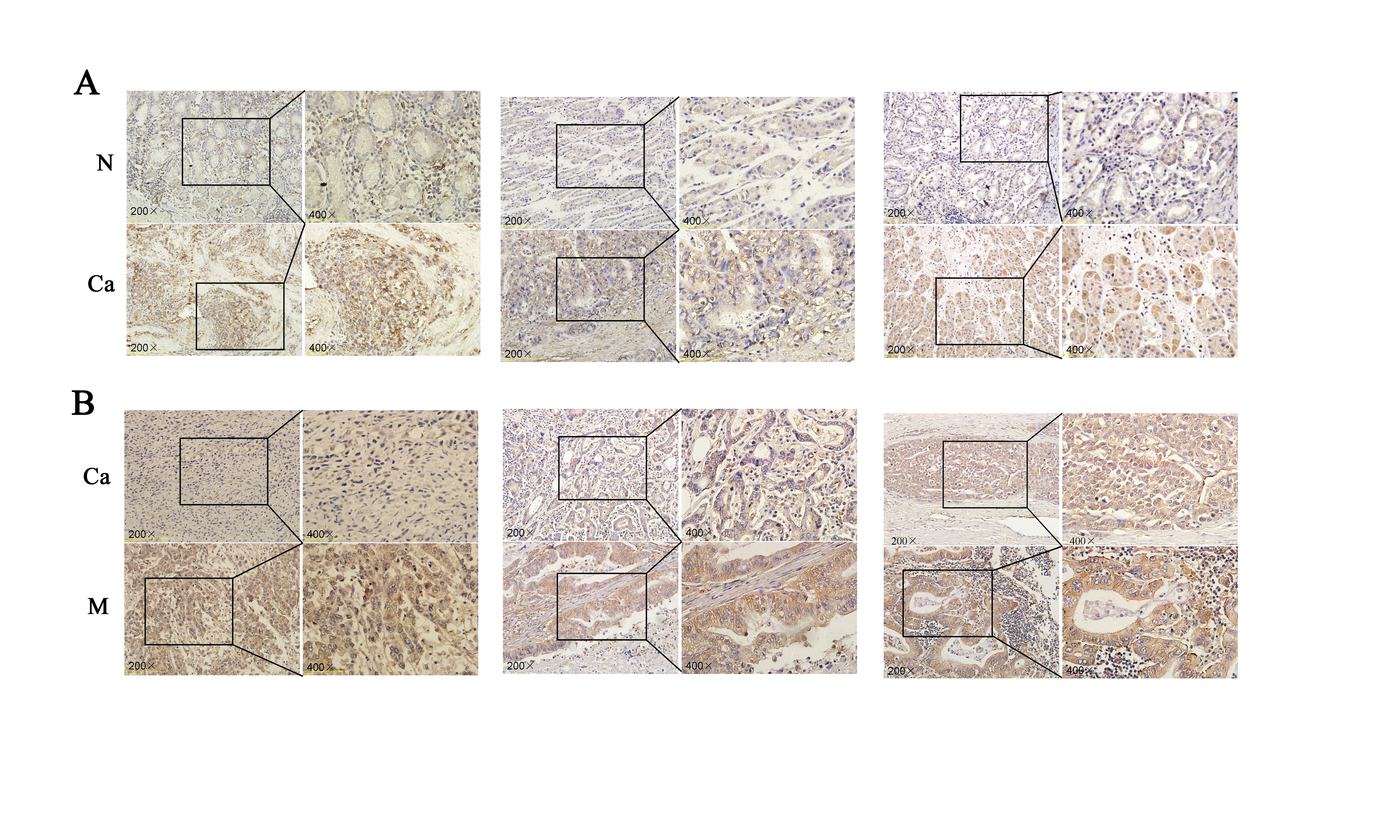

Supplement: Figure S1 — The expression of MYH9 in gastric tumor specimens. (A) Expression of MYH9 in primary gastric cancer (Ca) and its adjacent normal stomach tissue (N) by IHC. (B) Expression of MYH9 in primary gastric cancer (Ca) and its matched lymph node metastasis tissue (M) by IHC. (TIF) [file pone.0018409.s001.tif]
