# Supplementary material for: MicroRNA Let-7f Inhibits Tumor Invasion and Metastasis by Targeting MYH9 in Human Gastric Cancer
Source: PLoS One. 2011 Apr 18;6(4):e18409. doi: 10.1371/journal.pone.0018409 (PMC3078939; doi:10.1371/journal.pone.0018409)
Supplement: Table S1 — Clinicopathologic features in 8 fresh gastric tumor samples. (DOC) [file pone.0018409.s002.doc]

| NO | Age | Gender | N | Ca | M | TNM | Diffrentiation degree |
| --- | --- | --- | --- | --- | --- | --- | --- |
| 1 | 36 | Femal | 3×４×４cm  Normal gastric tissues | 4×6×4cm  gastric adenocarcinoma | 2×3×2cm  Lymph node（epiploon） | T_3_N_3_M_Ｘ_ | Poorly  differentiated |
| 2 | 44 | Femal | 4×3×3cm  Normal gastric tissues | 4×5×4cm  gastric adenocarcinoma | 3×3×3cm  Lymph node（epiploon） | T_3_N_3_M_Ｘ_ | Poorly  differentiated |
| 3 | 64 | Male | 3×3×3cm  Normal gastric tissues | 4×4×4cm  gastric adenocarcinoma | 4×3×2cm  Lymph node（cardia） | T_3_N_3_M_Ｘ_ | Moderately  differentiated |
| 4 | 55 | Male | 4×3×3cm  Normal gastric tissues | 5×5×3cm  gastric adenocarcinoma | 4×2×3cm  Lymph node  (lesser curvature of stomach） | T_3_N_２_M_Ｘ_ | Well  differentiated |
| 5 | 62 | Male | 5×4×4cm  Normal gastric tissues | 4×3×4cm  gastric adenocarcinoma | 3×3×3cm  Lymph node  （lesser curvature of stomach） | T_3_N_２_M_Ｘ_ | Poorly  differentiated |
| 6 | 59 | Femal | 4×3×5cm  Normal gastric tissues | 5×5×5cm  gastric adenocarcinoma | 2×2×2cm  Lymph node（greater curvature of stomach） | T_２_N_２_M_Ｘ_ | Moderately  and poorly-differentiated |
| 7 | 64 | Male | 2×3×2cm  Normal gastric tissues | 3×3×2cm  gastric adenocarcinoma | 2×1×2cm  Lymph node（greater curvature of stomach） | T_２_N_２_M_Ｘ_ | Moderately and poorly-differentiated |
| 8 | 60 | Male | 4×3×2cm  Normal gastric tissues | 4×4×3cm  gastric adenocarcinoma | 3×2×2cm  Lymph node（greater curvature of stomach） | T_3_N_２_M_Ｘ_ | Poorly  differentiated |

**Supplementary Table1.** Clinicopathologic features in 8 fresh gastric tumor samples (Real time PCR)
